# HDL-C Response Variability to Niacin ER in US Adults

**DOI:** 10.1155/2013/681475

**Published:** 2013-02-26

**Authors:** Jennifer B. Christian, Eric J. Olson, Jeffery K. Allen, Kimberly A. Lowe

**Affiliations:** ^1^GlaxoSmithKline, Clinical Effectiveness & Safety, 5 Moore Drive, B.3116, Durham, NC 27709-3398, USA; ^2^GlaxoSmithKline, Heart Failure DPU, Medicines Discovery and Development, King of Prussia, PA 19406, USA; ^3^GlaxoSmithKline, Worldwide Epidemiology, Durham, NC 27709, USA; ^4^Exponent Health Sciences, Bellevue, WA 98004, USA

## Abstract

*Background*. Niacin is the most effective treatment currently available for raising HDL-C levels. *Objective*. To evaluate if gender and baseline lipid levels have an effect on the HDL-C response of niacin ER and to identify factors that predict response to niacin ER at the 500 mg dose. *Material and Methods*. The change in HDL-C effect between baseline and follow-up levels was quantified in absolute change as well as dichotomized into high versus low response (high response was defined as an HDL-C effect of >15% increase and low response was HDL-C <5%) in a sample of 834 individuals. *Results*. Both males and females with low HDL-C levels at baseline exhibited a response to treatment in the multivariate model (males, HDL-C <40 mg/dL: OR = 5.18, 95% CI: 2.36–11.39; females, HDL-C <50 mg/dL: OR = 5.40, 95% CI: 1.84–15.79). There was also a significant difference in the mean HDL-C effect between baseline and follow-up HDL-C levels in the 500 mg niacin ER dose group for both males (mean HDL-C effect = 0.08, *P* < 0.001) and females (mean HDL-C effect = 0.10, *P* = 0.019). *Conclusion*. Baseline HDL-C levels are the biggest predictor of response to niacin ER treatment for both males and females among the factors evaluated.

## 1. Introduction 

High-density lipoprotein cholesterol (HDL-C), also referred to as the “good cholesterol,” is a known antioxidant, anti-thrombotic, and antiinflammatory, with properties that promote the removal of cellular cholesterol [1]. Individuals with low HDL-C levels may be at an increased risk of coronary heart disease (CHD) [2], and it has been shown in observational studies that the risk of CHD is reduced by 2%-3% for every 1 mg/dL increase in HDL-C [3]. Approximately 16% of adults in the United States have low HDL-C levels (25% of males and 7% of females) [4], while 11% of males and 4% of females of 19 years of age or younger have low HDL-C [5].

Low HDL-C is managed by three classes of dyslipidemic pharmacotherapy: niacin, statins, and fibric acid derivatives. Of these, niacin has been shown to increase HDL-C levels by as much as 35% in higher doses, which makes it the most effective pharmaceutical treatment currently available for raising HDL-C [6–9]. In addition, extended-release (ER) high-dose niacin has been shown to significantly reduce carotid atherosclerosis within 12 months among statin-treated individuals with low HDL-C who had either type II diabetes with coronary heart disease or carotid/peripheral atherosclerosis [9]. However, recently published results from the Atherothrombosis Intervention in Metabolic Syndrome with Low HDL Cholesterol/High Triglyceride and Impact on Global Health Outcomes (AIM-HIGH) study showed that high-dose niacin ER did not provide additional reduction in risk of cardiovascular-related events beyond what is observed with statin therapy alone [4]. In this modest sized trial, 3,414 subjects were randomized to niacin ER or placebo while their low-density lipoprotein cholesterol (LDL-C) levels were maintained to a median of <70 mg/dL. Importantly, the lipid community still awaits the results of a second larger niacin ER study (HSP2 THRIVE) to understand where the potential value of niacin therapy may reside in CV risk reduction, if it does at all. Therefore, due to the findings of the AIM-HIGH study and until the results of the HSP2 THRIVE results are revealed (in addition to the poor tolerability of niacin itself), administration of niacin to those who stand to achieve the most lipid benefit is important. 

Therefore, our objectives were to evaluate the extent to which gender and baseline lipid levels may have an effect on the HDL-C response of niacin ER and to identify factors that predict greater response to niacin ER.

## 2. Materials and Methods

### 2.1. Data Source and Description of the Study Population

This is a retrospective cohort study using medical claims that occurred between January 1, 2000, and March 31, 2008, from the Optum Impact National Managed Care Benchmark Database (OptumInsight, Eden Prairie, MN, USA). This database includes de-identified claims for inpatient and outpatient services, laboratory results, and pharmacy fills for privately insured patients, including several hundred thousand Managed Medicare and Medicare Advantage members. It is a commonly used and reliable data source for epidemiologic research in the United States [7, 9–12]. Database administrators perform multiple cross-checks to ensure data accuracy. Such practices include tracking of historical trends to identify anomalies and outliers. Database metrics are run after each update to cross-check the data and ensure it meets known population distributions. Source-to-target validation of data quantities is also completed and prescribed rules for researching and discarding nonconforming data are followed. The patients included in the database are representative of privately insured patients in all 50 states. A large proportion of the lab results come from two major lab vendors. 

The sample for the current study includes patients ≥18 years of age who met the following criteria: (1) concurrent medical and prescription coverage for the study period for at least two months while taking niacin ER, (2) a prescription of at least 28 days of niacin ER filled during the study period, (3) continuous eligibility for six months prior to the first niacin ER prescription and ≥29 days after the first prescription at the desired dose of niacin ER, (4) HDL-C measurements were available ≤6 months prior to and ≥29 days after filling the first prescription of niacin ER, and (5) gender was provided in the claims record.

### 2.2. Niacin ER Dose Levels

Prescription niacin ER was measured at 500 mg and 1000 mg doses. The two niacin ER dose groups were mutually exclusive, and all study participants were taking the niacin ER in combination with a statin. Individuals who qualified for the study in more than one dose group were categorized into the highest dose group for the purposes of this study. 

### 2.3. Baseline Lipid Levels

Baseline lipid levels were taken within a six-month period prior to starting niacin ER at any dose and were dichotomized into the following groups for analysis: HDL-C levels for males and females combined (<40 mg/dL versus ≥40 mg/dL), HDL-C levels for males only (HDL-C<40 mg/dL versus ≥40 mg/dL), HDL-C levels for females only (HDL-C <50 mg/dL versus ≥50 mg/dL), LDL-C levels (<130 mg/dL versus ≥130 mg/dL), triglyceride (TG) levels <150 mg/dL versus ≥150 mg/dL), and total cholesterol (TC) levels (<200 mg/dL versus ≥200 mg/dL). 

### 2.4. Change in HDL Effect

Follow-up HDL-C levels were included in the analysis after the first month of treatment with niacin ER (at the specific dose of interest) through 5 days after termination of niacin ER. The distribution of change in HDL-C effect was evaluated with 5% intervals by gender and also by dose of niacin ER. For the purposes of statistical analysis, the change in HDL-C effect was measured in two ways: (1) as a continuous variable and (2) dichotomized into high versus low response, in which high response was defined as an HDL-C effect of >15% and low response was HDL-C <5%. These cut points were selected based on a combination of the distribution of HDL-C response in our sample and a meaningful clinical response to niacin ER. 

### 2.5. Statistical Analysis

Baseline demographic characteristics and lipid levels were summarized as frequencies and percentages. The change in HDL-C effect between baseline and followup was quantified by the difference and divided by the baseline value and then summarized using mean values and standard deviations. The Student's *t*-test was used to test the null hypothesis that there was no difference in the mean HDL-C effect by dichotomous categorical variables. The *F*-statistic was used to test the null hypothesis that there was no difference in the mean HDL-C effect across variables with three or more categories. Crude and adjusted multivariate logistic regression models were then developed to evaluate HDL-C response to niacin ER at the 500 mg dose level by calculating an odds ratio (OR) and 95% confidence interval (CI). As described above, our main analysis defined high responders as individuals with an HDL-C effect of >15% and low responders were defined as individuals with a <5% HDL-C effect. The outcomes in these models were high response (yes or no). 

A sensitivity analysis was also conducted where high response was defined as a combination of HDL-C rise of >15% and LDL-C lowering effect of >−15%, and low response was defined as <5% HDL-C effect and LDL-C effect was >−15%. This restricted definition was used to ensure that subjects were taking their combination medications of niacin and a statin but not achieving the anticipated rise in HDL-C. Crude and adjusted multivariate logistic regression models were developed using this restricted definition to evaluate HDL-C response to niacin ER at the 500 mg dose level. 

## 3. Results

Our sample included a total of 834 study participants (680 at the 500 mg niacin ER dose level, and 154 at the 1000 mg niacin ER dose level).  Table 1 summarizes the baseline characteristics and lipid levels of the study participants by niacin ER dose level. The majority of study participants in each niacin ER dose level were 41 to 65 years of age, male, had HDL-C levels <40 mg/dL, had baseline LDL-C levels <130 mg/dL, and had baseline triglyceride levels ≥150 mg/dL. The majority of study participants in the 500 mg niacin ER dose group had baseline total cholesterol levels ≥200 mg, while the majority of the study participants in the 1000 mg niacin ER dose group had baseline total cholesterol levels <200 mg/dL. The proportion of study participants who had diabetes, stroke (within six months), hypertension (within six months), and ischemic heart disease (within six months) were similar in both dose groups. The average persistence of niacin ER was 193.6 days in the 500 mg dose group and 192.1 in the 1000 mg dose group.

**Figure 1:**
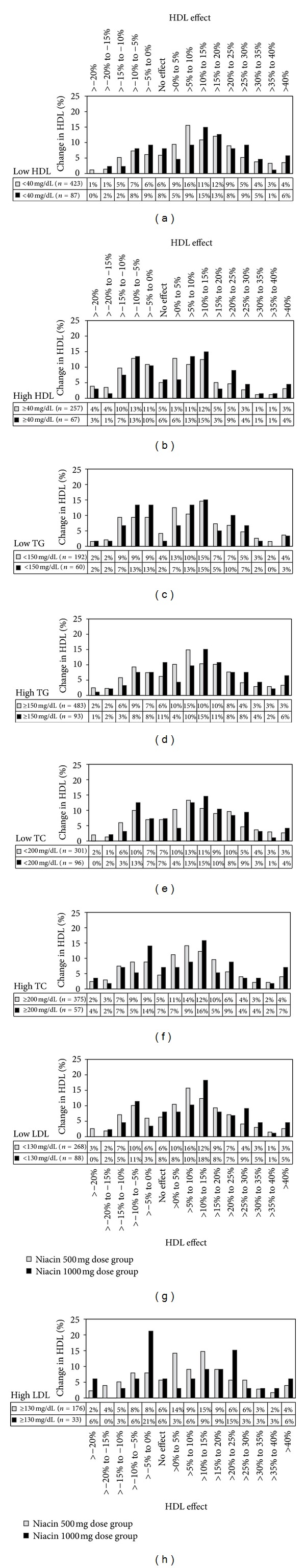
HDL effect by niacin ER dose and baseline lipid levels: (a) low HDL (<40 mg/dL), (b) high HDL (>40 mg/dL), (c) low TG (<150 mg/dL), (d) high TG (>150 mg/dL), (e) low TC (<200 mg/dL), (f) high TC (>200 mg/dL), (g) low LDL (<130 mg/dL), and (h) high LDL (≥130 mg/dL).

Figure 1 illustrates the distribution of HDL-C effect by niacin ER dose and baseline lipid levels, and Table 2 reports the results of the inferential statistics of the overall HDL-C effect by niacin ER dose. There was a statistically significant change in the mean HDL-C effect between baseline and follow-up HDL-C levels in the 500 mg niacin ER dose group for both males (mean HDL-C effect = 0.08, *P* < 0.001) and females (mean HDL-C effect = 0.10, *P* = 0.019). Similar results were observed in the 1000 mg niacin ER dose group for males (mean HDL-C effect = 0.08, *P* = 0.029) and females (mean HDL-C effect = 0.19, *P* = 0.048). Females had statistically significantly higher levels of both baseline and follow-up HDL-C levels than males in both niacin ER dose groups. In addition, there was a statistically significant difference in the HDL-C effect observed among females across the niacin ER dose groups (*P* = 0.021). This trend was not observed among males (*P* = 0.768). There was a statistically significant change in the mean HDL-C effect between baseline and follow-up HDL-C levels in both niacin ER dose groups among individuals of 41 to 65 years of age (mean HDL-C effect in the 500 mg dose group = 0.08, *P* < 0.001; mean HDL-C effect in the 1000 mg dose group = 0.12, *P* = 0.008), but not among individuals in the 18 to 40 or ≥66 age groups. The mean HDL-C levels were statistically significantly higher in the >66-year age group than the other age groups at baseline (*P* = 0.03) and followup (*P* = 0.018) among participants in the 500 mg niacin ER dose group but not in the 1000 mg niacin ER dose group. The change in the mean HDL-C effect between baseline and follow-up HDL-C levels was statistically significant for individuals with HDL-C levels <40 mg/dL in the 500 mg niacin ER dose group (mean HDL-C effect = 0.12, *P* < 0.001) and also for individuals in the 1000 mg niacin ER dose group (mean HDL-C effect = 0.13, *P* < 0.001). There was no change in the HDL-C effect among individuals with HDL-C levels ≥40 mg/dL in either niacin ER dose group. When baseline LDL-C levels were stratified at 130 mg/dL, there was a statistically significant difference in the mean HDL-C effect between baseline and follow-up HDL-C levels among individuals who had LDL-C levels <130 mg/dL (mean HDL-C effect = 0.07, *P* = 0.025) and among those who had LDL-C ≥130 (mean HDL-C effect = 0.08, *P* = 0.031) in the 500 mg niacin ER dose. Among individuals in the 1000 mg niacin ER dose group, only individuals with LDL-C levels <130 showed a statistically significant improvement in the mean HDL-C effect (mean HDL-C effect = 0.12, *P* = 0.008). The mean HDL-C effect was statistically significantly higher among individuals with triglyceride levels ≥150 mg/dL (mean HDL-C effect = 0.08, *P* < 0.001) in the 500 mg niacin ER dose group and among individuals in that TG category in the 1000 mg niacin ER dose group (HDL-C effect = 0.12, *P* = 0.005). There were no significant changes in the HDL-C effect in either niacin ER dose group among individuals with triglyceride levels <150 mg/dL. There was a statistically significant difference in the mean HDL-C effect among individuals with total cholesterol levels <200 mg/dL (mean HDL-C effect = 0.09, *P* = 0.001) and among individuals with total cholesterol levels ≥200 mg/dL (mean HDL-C effect = 0.08, *P* = 0.004) in the 500 mg niacin ER dose group, but only among individuals with total cholesterol levels <200 mg/dL in the 1000 mg niacin ER dose group (mean HDL-C effect = 0.011, *P* = 0.007). 

Table 3 summarizes the results of the univariate and multivariate logistic regression models that evaluated the predictors of high response to the 500 mg dose of niacin ER. The only statistically significant predictors of response to niacin ER in the univariate models were triglyceride levels >150 mg/dL and low HDL-C levels (<40 mg/dL for males and <50 mg/dL for females). Triglyceride levels did not maintain statistical significance in the multivariate model. However, both males and females with low HDL-C levels exhibited a strong response to treatment in the multivariate model (males, HDL-C <40 mg/dL: OR = 5.18, 95% CI: 2.36–11.39; females, HDL-C <50 mg/dL: OR = 5.40, 95% CI: 1.84–15.79). Similar, but attenuated, trends were observed in the multivariate sensitivity analysis model (males, HDL-C <40 mg/dL: OR = 4.90, 95% CI: 1.56–15.46; females, HDL-C <50 mg/dL: OR = 2.34, 95% CI: 0.61–9.04) (data not shown). 

## 4. Discussion

Approximately one-quarter of the adult population in the USA has low HDL-C [8]. The causes of low HDL-C levels are largely unknown; however, there is evidence to suggest that being overweight or obese, having limited physical activity, smoking, increased intake of carbohydrates, Type II diabetes, genetic factors, and the use of some medications (i.e., beta-blockers, anabolic steroids, and progestational agents) may increase the likelihood of having low HDL-C [13]. A recent evaluation of data from the National Health and Nutrition Examination Survey (NHANES) identified several factors that were associated with HDL-C levels among *both* men and women. Specifically, being obese, having high apolipoprotein B levels (>117 mg/dL), and having high triglyceride levels (≥200 mg/dL) were shown to be associated with low HDL-C levels, while increased age (65 years or older) and having elevated total cholesterol (>240 mg/dL) were shown to be associated with elevated HDL-C levels [4]. 

The clinical management of HDL-C is a somewhat vexing topic for several reasons. First, treatment with dyslipidemic pharmacotherapy, primarily niacin, has been shown to increase HDL-C levels by as much as 35% in some populations [6–9]. However, the manner in which these positive increases translate into reduced morbidity and mortality has been disappointing in recent studies. Results from the AIM-HIGH study showed a lack of efficacy in reducing cardiovascular-related events after 3.5 years, which led to the early termination of this study [4]. Second, niacin is known to cause cutaneous vasodilatation with flushing, which may include itching, tingling, warmth, and redness, which can lead to poor adherence or early discontinuation [14, 15]. A cohort study of 2,369 individuals who were newly prescribed dyslipidemic agents reported a one-year probability of discontinuation of 46% for niacin versus 15% for lovastatin [4, 16].

Overall, our results showed that treatment with niacin ER at both the 500 mg and 1000 mg dose levels significantly increased HDL-C levels in both men and women. However, the average increase we observed was much lower than the 35% increase that has been observed in some populations. In our study, females had significantly higher levels of both baseline and follow-up HDL-C levels than males in both niacin ER dose groups. Furthermore, there was a statistically significant difference in the HDL-C effect observed among females across the niacin ER dose groups. This trend was not observed among males. When the association between age and effect of treatment was evaluated, the only statistically significant change in the mean HDL-C effect between baseline and follow-up HDL-C levels in both niacin ER dose groups was among individuals of 41 to 65 years of age. The mean HDL-C levels were statistically significantly higher in the >66-year age group compared with the younger age groups at both baseline and follow-up HDL-C measurements among participants in the 500 mg niacin ER dose group but not in the 1000 mg niacin ER dose group. 

It is because of these paradoxical associations that we sought to identify factors that predict response to niacin ER, with the goal of providing useful information that may lead to the most efficient and successful prescription patterns for this treatment. Unfortunately, we were not able to conduct univariate and multivariate models for those treated with niacin ER at the 1000 mg dose due to a smaller sample size. However, we did find that baseline HDL-C levels were the only factor that significantly predicted a high response to treatment with niacin ER at the 500 mg dose level in the multivariate models. These results provide some insight into the factors that are associated with the effect of niacin treatment. 

Limitations of our study may include the manner in which high response to niacin ER was classified. High response was defined as an HDL-C effect of >15% and low response was defined as <5% HDL-C effect. These thresholds were selected based on the distribution of response in our sample irrespective of characteristics and determined to be clinically meaningful responses. Modifications in these thresholds may have resulted in a slight change in our findings. However, we conducted a sensitivity analysis among people who were responding to taking their medications by including only those who had an LDL-C decrease of 15% or greater and our findings were consistent. Although monitoring prescription fills and biomarker response is not identical to consuming the medication, this method was the most accurate way of estimating actual medication use in a large population. In addition, clinical variables such as body mass index, exercise, diet, and smoking were not available in this healthcare claims database. We did not include other medications that may control HDL-C levels, such as statins, in our models. However, the majority of the patients included in this study were taking simvastatin at 20 mg or 40 mg doses. There is evidence to suggest that statins (as a drug class) only modestly increase HDL-C levels, with less than a 10% increase reported in two studies [16, 17]. Finally, the quality control of laboratory measurements is assumed but cannot be verified. The laboratory values provided in the database are those that were provided by the laboratory vendor. As such, the specific laboratory methods or reference ranges that correspond with the lipid values are not available. Despite these limitations, the strengths of this study include the large sample size, which allowed us to evaluate a highly stratified matrix of response to treatment. 

In summary, individuals with low HDL-C levels at baseline may have an increased risk of CHD-related events; however, the issues surrounding the pharmacological management of HDL-C are complex. We have shown that low baseline HDL-C levels are the strongest predictor of high response from the treatment of niacin ER at the 500 mg dose level, among statin-treated individuals. We have also shown that both men and women had a significant increase in the mean HDL-C effect between baseline and followup, regardless of niacin ER dose. These findings can help clinicians and researchers better understand the extent to which response to niacin ER is variable and the importance of baseline HDL-C influencing response size while further data is generated to elucidate the value of HDL-C-raising therapy. 

## Figures and Tables

**Table 1:** Baseline characteristics and lipid levels^1^.

Demographics and lipids	500 mg niacin ER dose (*n* = 680)	1000 mg niacin ER dose (*n* = 154)
	*n* (%)	*n* (%)
Age (years)		
18 to 40	79 (11.6)	12 (7.8)
41 to 65	516 (75.9)	131 (85.1)
≥66	85 (12.5)	11 (7.1)
Gender		
Male	480 (70.6)	120 (77.9)
Female	200 (29.4)	34 (22.1)
HDL-C mg/dL (both genders)		
<40	419 (61.6)	87 (56.5)
40 to 59	213 (31.3)	59 (38.3)
≥60	44 (6.5)	8 (5.2)
Baseline LDL-C mg/dL		
<130	266 (39.1)	87 (56.5)
≥130	178 (26.2)	34 (22.1)
Unknown	236 (34.7)	33 (21.4)
Baseline TG mg/dL		
<150	192 (28.2)	60 (39.0)
≥150	483 (71.0)	93 (60.4)
Unknown	5 (0.7)	1 (0.7)
Baseline TC mg/dL		
<200	301 (44.3)	96 (62.3)
≥200	375 (55.2)	57 (37.0)
Unknown	4 (0.6)	1 (0.7)
Comorbidities		
Diabetes	188 (27.7)	41 (26.6)
Stroke (within 6 months)	26 (3.8)	10 (6.5)
Hypertension (within 6 months)	46 (6.8)	7 (4.6)
Ischemic heart disease (within 6 months)	130 (19.1)	32 (20.8)
Medication use		
Prestatin users	258 (37.9)	64 (41.6)
Prefibric acid users	82 (12.1)	12 (7.8)
Persistence		
Average persistence (days)	193.6	192.1
≥365 days persistence	79 (11.6)	17 (11.0)
<365 days persistence	601 (88.4)	137 (89.0)

^1^Lipid levels were measured within six months prior to any niacin ER use. Definition of lipids: HDL-C: high-density lipoprotein cholesterol; LDL-C: low-density lipoprotein cholesterol; TG: triglycerides; TC: total cholesterol.

**Table 2:** Overall HDL effect by niacin ER dose.

Demographics and lipids	500 mg niacin ER dose					1000 mg niacin ER dose					*P* value^b^
	*n*	Baseline HDL-C levels Mean (SD)	Follow-up HDL-C levels Mean (SD)	HDL-C effect Mean (SD)	*P* value^a^	*n*	Baseline HDL-C levels Mean (SD)	Follow-up HDL-C levels Mean (SD)	HDL-C effect Mean (SD)	*P* value^a^
Gender											
Males	480	37.1 (0.4)	39.4 (0.4)	0.08 (0.01)	<0.001	120	39.0 (0.9)	41.6 (0.9)	0.08 (0.02)	0.029	0.768^b^
Females	200	45.5 (1.1)	49.3 (1.2)	0.10 (0.01)	0.019	34	48.5 (2.6)	57.4 (3.6)	0.19 (0.03)	0.048	0.021^b^
*P* value^c^		<0.001	<0.001	0.221			0.001	<0.001	0.009		0.053^d^
Age (years)											
18 to 40	79	37.9 (1.2)	40.0 (1.1)	0.10 (0.04)	0.196	12	38.9 (2.9)	40.4 (1.7)	0.07 (0.05)	0.659	0.660^b^
41 to 65	516	39.4 (0.5)	42.2 (0.6)	0.08 (0.01)	<0.001	131	41.3 (1.1)	45.8 (1.3)	0.12 (0.02)	0.008	0.057^b^
≥66	85	42.5 (1.4)	45.5 (1.4)	0.08 (0.02)	0.126	11	41.2 (2.2)	41.9 (1.8)	0.03 (0.05)	0.803	0.335^b^
*P* value^d^		0.032	0.018	0.842			0.799	0.343	0.242		0.613^d^
HDL-C mg/dL (both genders)											
40	423	32.8 (0.3)	36.3 (0.4)	0.12 (0.01)	<0.001	87	33.5 (0.5)	37.7 (0.8)	0.13 (0.02)	<0.001	0.437^b^
≥40	257	50.8 (0.7)	52.3 (0.8)	0.03 (0.01)	0.168	67	50.9 (1.2)	54.7 (1.9)	0.07 (0.02)	0.096	0.094^b^
*P* value^c^		<0.001	<0.001	<0.001			<0.001	<0.001	0.037		0^d^
LDL-C mg/dL											
<130	264	39.0 (0.7)	41.3 (0.8)	0.07 (0.01)	0.025	88	40.6 (1.1)	44.9 (1.2)	0.12 (0.02)	0.008	0.160^b^
≥130	176	41.8 (0.9)	44.6 (1.0)	0.08 (0.02)	0.031	33	44.7 (2.7)	48.9 (3.5)	0.09 (0.03)	0.344	0.802^b^
*P* value^c^		0.011	0.006	0.474			0.161	0.291	0.489		0.472^d^
TG mg/dL											
<150	192	44.0 (1.1)	46.5 (1.1)	0.08 (0.02)	0.102	60	46.6 (1.6)	50.4 (2.2)	0.08 (0.02)	0.166	0.899^b^
≥150	483	38.0 (0.4)	40.9 (0.5)	0.08 (0.01)	<0.001	93	37.6 (1.0)	41.8 (1.1)	0.12 (0.02)	0.005	0.060^b^
*P* value^c^		<0.001	<0.001	0.943			<0.001	0.001	0.111		0.362^d^
TC mg/dL											
<200	301	36.8 (0.6)	39.5 (0.6)	0.09 (0.01)	0.001	96	39.6 (1.0)	43.7 (1.2)	0.11 (0.02)	0.007	0.362^b^
≥200	375	42.2 (0.6)	44.9 (0.7)	0.08 (0.01)	0.004	57	43.7 (1.9)	47.6 (2.4)	0.10 (0.03)	0.200	0.530^b^
*P* value^c^		<0.001	<0.001	0.259			0.052	0.144	0.549		0.195^d^

Definition of lipids: HDL-C: high-density lipoprotein cholesterol; LDL-C: low-density lipoprotein cholesterol; TG: triglycerides; TC: total cholesterol.

^a^ *P* value tested the null hypothesis that there is no difference in the baseline and follow-up HDL-C levels using the Student's *t*-test.

^b^ *P* value tested the null hypotheses that there is no difference in the mean HDL-C effect across niacin ER dose groups using the Student's *t*-test.

^c^ *P* value tested the null hypothesis that there is no difference in the mean HDL-C effect across each risk factor/lipid level category using the Student's *t*-test.

^d^ *P* value tested the null hypothesis that there is no difference in the mean HDL-C effect across each risk factor/lipid level category using the *F*-statistic.

**Table 3:** Predictors of response to niacin ER (500 mg dose).

	High responder^1^ (*n* = 202)	Low responder^2^ (*n* = 305)	Crude OR (95% CI)^3^	Adjusted OR (95% CI)^3,4^
	*n* (%)	*n* (%)		
Age (years, continuous)	—	—	0.10 (0.97–1.02)	1.0 (0.97–1.03)
Gender				
Female	39 (32.5%)	53 (29.1%)	1.0	1.0
Male	81 (67.5%)	129 (70.9%)	0.85 (0.52–1.40)	0.79 (0.25–2.51)
HDL-C mg/dL (males only)				
≥40	49 (40.8%)	111 (61.0%)	1.0	1.0
40	71 (59.2%)	71 (39.0%)	2.27 (1.42–3.63)	5.18 (2.36–11.39)
HDL-C mg/dL (females only)				
≥50	87 (72.5%)	155 (85.2%)	1.0	1.0
<50	33 (27.5%)	27 (14.8%)	2.18 (1.23–3.86)	5.40 (1.84–15.79)
LDL-C mg/dL				
<130	69 (57.5%)	103 (56.6%)	1.0	1.0
≥130	51 (42.5%)	79 (43.4%)	0.96 (0.61–1.54)	1.23 (0.54–2.77)
TG mg/dL				
<150	32 (26.7%)	71 (39.0%)	1.0	1.0
≥150	88 (73.3%)	111 (61.0%)	1.76 (1.06–2.91)	1.38 (0.76–2.50)
TC mg/dL				
<200	62 (51.7%)	85 (46.7%)	1.0	1.0
≥200	58 (48.3%)	97 (53.3%)	0.82 (0.52–1.30)	1.0 (0.42–2.34)
Diabetes				
No	82 (68.3%)	138 (75.8%)	1.0	1.0
Yes	38 (31.7%)	44 (24.2%)	1.45 (0.87–2.43)	1.41 (0.78–2.52)
Stroke (within 6 months)				
No	114 (95.0%)	174 (95.6%)	1.0	1.0
Yes	6 (5.0%)	8 (4.4%)	1.15 (0.39–3.39)	0.69 (0.19–2.49)
Hypertension (within 6 months)				
No	108 (90.0%)	171 (94.0%)	1.0	1.0
Yes	12 (10.0%)	11 (6.0%)	1.73 (0.74–4.05)	1.91 (0.71–5.15)
Ischemic heart disease (within 6 months)				
No	97 (80.8%)	147 (80.8%)	1.0	1.0
Yes	23 (19.2%)	35 (19.2%)	1.0 (0.56–1.79)	0.91 (0.44–1.87)
Statin use during baseline				
No	50 (41.7%)	63 (34.6%)	1.0	1.0
Yes	70 (58.3%)	119 (65.4%)	0.74 (0.46–1.19)	0.69 (0.37–1.31)
Fibric acid use during Baseline				
No	10 (8.3%)	22 (12.1%)	1.0	1.0
Yes	110 (91.7%)	160 (87.9%)	1.51 (0.69–3.32)	1.60 (0.69–3.75)

Definition of lipids: HDL-C: high-density lipoprotein cholesterol; LDL-C: low-density lipoprotein cholesterol; TG: triglycerides; TC: total cholesterol.

^1^High responder: HDL-C effect > 15%.

^2^Low responder: HDL-C effect < 5%.

^3^Outcome is high responder.

^4^Adjusted model includes all variables listed in the table.
